# Anti-Interleukin-1 Therapy Does Not Affect the Response to SARS-CoV-2 Vaccination and Infection in Patients with Systemic Autoinflammatory Diseases

**DOI:** 10.3390/jcm12247587

**Published:** 2023-12-08

**Authors:** Leonie Geck, Koray Tascilar, David Simon, Arnd Kleyer, Georg Schett, Jürgen Rech

**Affiliations:** 1 Department of Internal Medicine 3, Rheumatology and Immunology, Universitätsklinikum Erlangen, Friedrich-Alexander-University Erlangen-Nürnberg (FAU), 91054 Erlangen, Germany; leonie.geck@fau.de (L.G.); koray.tascilar@uk-erlangen.de (K.T.); simon.david@extern.uk-erlangen.de (D.S.); arnd.kleyer@extern.uk-erlangen.de (A.K.); georg.schett@uk-erlangen.de (G.S.); 2 Deutsches Zentrum Immuntherapie, Universitätsklinikum Erlangen, Friedrich-Alexander-University Erlangen-Nürnberg (FAU), 91054 Erlangen, Germany; 3 Centre for Rare Diseases Erlangen, Universitätsklinikum Erlangen, Friedrich-Alexander-University Erlangen-Nürnberg (FAU), 91054 Erlangen, Germany

**Keywords:** autoinflammatory diseases, SARS-CoV-2, vaccination, familial Mediterranean fever, adult-onset Still’s disease, cryopyrin-associated periodic syndrome, systemic autoinflammatory diseases

## Abstract

Patients with systemic autoinflammatory diseases (sAIDs) are a section of the population at high risk of severe COVID-19 outcomes, but evidence on the efficacy of SARS-CoV-2 vaccination in this group of patients is scarce. To investigate the efficacy of SARS-CoV-2 vaccination in patients with sAIDs receiving interleukin-1 (IL-1) inhibition is important. Vaccination and infection responses from 100 sAID patients and 100 healthy controls (HCs) were analyzed. In total, 98% of patients were treated with IL-1 inhibitors at the time of vaccination (*n* = 98). After the second SARS-CoV-2 vaccination, sAID patients showed similar anti-SARS-CoV-2 antibody responses (mean (standard deviation (SD)): 6.7 (2.7)) compared to HCs (5.7 (2.4)) as well as similar neutralizing antibodies (85.1 ± 22.9% vs. 82.5 ± 19.7%). Anti-SARS-CoV-2 antibody responses and neutralizing antibodies were similar in sAID patients after SARS-CoV-2 infection and double vaccination. Furthermore, while antibodies increased after the first and second vaccination in sAID patients, they did not further increase after the third and fourth vaccination. No difference was found in antibody responses between anakinra and anti-IL-1 antibody treatment and the additional use of colchicine or other drugs did not impair vaccination responses. Primary and booster SARS-CoV-2 vaccinations led to protective antibody responses in sAID patients, which were at the same level of vaccination responses in HCs and in sAID patients after SARS-CoV-2 infection. Immunomodulatory treatments used in sAID do not seem to affect antibody responses to the SARS-CoV-2 vaccine.

## 1. Background

Immune responses to vaccines depend on the quality of the vaccine used and the immune response of the host. In immune-mediated inflammatory diseases (IMIDs), the immune response of the host is altered due to intrinsic changes in the innate and adaptive immune system as well as the concomitant presence of immune-modulatory drugs [1]. Initial studies on the use of SARS-CoV-2 vaccines in patients with IMIDs were reassuring as to the efficacy of the vaccines despite the use of different immune-modulatory drugs such as cytokine inhibitors [2], nevertheless indicating a reduced longevity in overall humoral responses to SARS-CoV-2 vaccines [3]. However, patients with systemic autoinflammatory diseases (sAIDs) were not included or were highly underrepresented in these studies, which substantially limits our current knowledge on the efficacy of SARS-CoV-2 vaccines in these disease groups [4]. Notably, sAID patients receive different drugs than other patient groups from the IMID disease spectrum. Thus, interleukin-1 (IL-1) inhibitors and colchicine are widely used to treat sAIDs. As commonalities between sAIDs and COVID-19 infection have been observed, e.g., dysfunctional cytokine release [5], these medications have been used outside the sAID field in the treatment of COVID-19, but with little to no effect on adult hospitalized patients [6,7]. Furthermore, under certain circumstances, such as comorbidities and glucocorticoid use, sAID patients are at risk of developing severe COVID-19 [8]. This observation indicates that better knowledge about the response of sAID patients to SARS-CoV-2 vaccine is of utmost importance.

We therefore addressed anti-SARS-CoV-2 antibody responses in a larger group of sAID patients treated with IL-1 inhibitors to find out whether sAID patients are able to mount sufficient humoral immunity against the coronavirus. We thereby analyzed primary antibody responses and booster responses and compared them to data obtained from healthy controls (HCs). In addition, we analyzed whether the additional use of colchicine influenced the vaccination response.

## 2. Materials and Methods

### 2.1. Participants

After approval by the Research Ethics Committee of the Friedrich-Alexander-University Erlangen-Nürnberg (FAU), 100 consecutive sAID patients were recruited. The HC group was age- and gender-matched with the large COVID-19 study at the Deutsche Zentrum Immuntherapie (DZI), Universitätsklinikum Erlangen (UKER), established in February 2020. Patient demographic data (age, sex, body mass index, comorbidities, medication) and information on SARS-CoV-2 vaccination (date, type of vaccine) and infection (date, general symptoms, severity of symptoms) were obtained through telephone interviews and supplemented using a retrospective analysis of physician letters in the UKER electronic archive and document management system Soarian^®^ (Soarin Clinicals, version 4.5.200, Bay Lake, FL, USA) until the end of July 2022.

### 2.2. Anti-SARS-CoV-2 Antibody Testing

Quantitative detection of specific antibodies of the IgG class against the SARS-CoV-2 spike protein was performed with the CE-marked version of the enzyme-linked immunosorbent assay (ELISA) kit from EUROIMMUN (Lübeck, Germany). The reagent vials were evaluated photometrically at an optical density (OD) of 450 nm with reference wavelength at 630 nm. The ratio was then determined by dividing the absorbances of the control or patient sample and the calibrator; a ratio ≥ 0.8 was considered positive.

The determination of SARS-CoV-2 neutralizing antibodies was performed with the CE-In Vitro Diagnostics (CE-IVD)-certified cPass surrogate virus neutralization assay from the manufacturer GenScript (Piscataway, NJ, USA). Per test run, neutralizing antibodies from 92 participants’ sera can be determined on a microtiter plate with two negative and two positive controls. Photometric analysis was performed at 450 nm and a calculation formula was used to determine the inhibition percentage. A cut-off value of 30% was considered a positive test result and indicated the presence of neutralizing antibodies.

### 2.3. Statistical Analysis

Characteristics of the study groups were described using descriptive analyses—including mean, standard deviation (SD) and counts/percentages. We used the Wilcoxon rank-sum test to compare controls with the sAID group. Since on average, a longer time had elapsed between the second vaccination and sample collection, we used linear regression to adjust for this time difference. Separate models were fitted for the antibody and neutralizing antibody values as independent variables and OD ratio values from the antibody assay as the dependent variables. The visual image of an initial scatter plot in the analysis indicated that the use of a native scale did not adequately present the data, which is why the neutralizing antibodies were analyzed in the logit transformation and the SARS-CoV-2 antibody ratio in the log transformation. The freely available programming language “R v.4.01” (R Foundation for Statistical Computing, Vienna, Austria) was used for all statistical analyses. A *p*-value < 0.05 was considered statistically significant.

## 3. Results

### 3.1. Characteristics of Patients and Controls

A total of 100 sAID patients were analyzed, of whom 58 were female and 42 were male (Table 1). At the time of data collection in June and July 2022, the age (mean (min; max) ± SD) of sAID patients was 43 (18; 80) ± 16 years. The largest proportionally represented disease was familial Mediterranean fever (FMF), present in more than one third (37%) of the patients, followed by adult-onset Still’s disease (AoSD; 25%), gout (15%), cryopyrin-associated periodic syndrome (CAPS; 7%), sAID of unclear etiology (7%), Behçet’s disease (4%), tumor necrosis receptor-associated periodic syndrome (TRAPS; 4%) and Yao syndrome (1%).

At the time of vaccination, 48 patients were treated with an IL-1 receptor antagonist (IL1RA) and 50 patients with an IL-1 inhibiting antibody, while two patients did not receive anti-IL1 treatment due to being in remission during the vaccination period. Three patients on treatment with anakinra received the IL1RA only on demand during a disease flare, whereas the remaining 95 patients received anti-IL1 treatment permanently. The mean dose was 104.17 mg (on average 6.6/days per week) for anakinra and 183 mg (once every 5.7 weeks) for canakinumab. The mean treatment duration before the first vaccination was 715 (SD: 1287) days for anakinra and 337 (511) days for canakinumab.

In addition to IL-1 inhibitors, colchicine was used in 36% of sAID patients. Glucocorticoids and methotrexate were infrequently used treatments. Four patients were treated with a dose of glucocorticoids lower than 10 mg per day, while one patient received a dose of 20 mg/day. By the end of July 2022, one patient had received only one shot of the COVID-19 vaccine, while 13 patients (13%) had received two, the vast majority (73%) had received three and 9% had received four vaccinations. Only four patients (4%) received no SARS-CoV-2 vaccination. In sAID patients, mRNA-based vaccines were by far the most frequently used, whereas only a minority of patients received a vector-based vaccine. The HC group (*n* = 100) had a similar age and sex distribution as the sAID patients and consisted of 57 females and 43 males with a mean age of 43 (18; 89) ± 16 years.

### 3.2. Anti-SARS-CoV-2 IgG and Neutralizing Antibodies Responses in sAID

**Figure 1:**
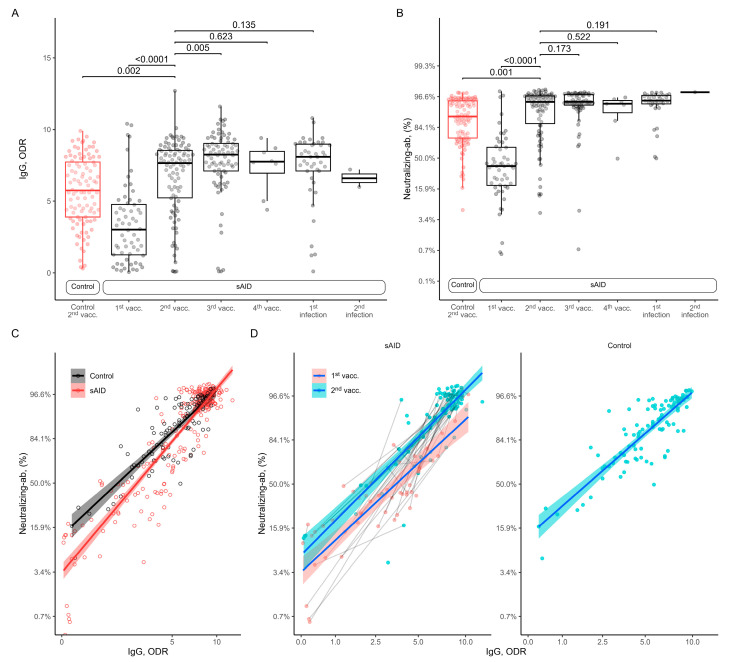
Association between anti-SARS-CoV-2 IgG and neutralizing antibodies in patients and healthy controls after COVID-19 vaccination and infection. (**A**) Anti-SARS-CoV-2 IgG antibodies and (**B**) SARS-CoV-2 neutralizing antibodies for sAID patients and HCs after COVID-19 vaccination and infection. (**C**) Anti-SARS-CoV-2 IgG and neutralizing antibodies in patients and HCs for all measured events. (**D**) Anti-SARS-CoV-2 IgG and neutralizing antibodies observed separately in patients after primary vaccination and in HCs after 2nd vaccination only. HC, healthy control; IgG (here), anti-SARS-CoV-2 IgG antibodies; Neutralizing-ab, neutralizing antibodies; ODR, optical density ratio; sAID, systemic autoinflammatory disease; vacc., vaccination.

All analyses of anti-SARS-CoV-2 IgG antibodies in sAID patients showed an increase from the first to the second vaccination (mean values in Table 2), with similar antibody levels after the second vaccination in sAID patients compared to HCs (Figure 1A). Further vaccinations did not increase anti-SARS-CoV-2 IgG antibody responses. Also, anti-SARS-CoV-2 IgG antibody levels after additional SARS-CoV-2 infection were comparable to the ones found after vaccination. The fact that HCs (5.7 (SD: 2.4)) had even lower anti-SARS-CoV-2 IgG levels than sAID patients (7 (2.7); *p* = 0.0017) after two vaccinations was due to the timing of sample collection after the second vaccination, which was 9.1 (7.5) weeks in the sAID group as compared to 21.9 (6.5) weeks in the control group. When we adjusted the sample collection time after the second vaccination, no difference in antibody levels between the HC and the sAID groups was found (adjusted mean between group difference 0.17; 95% CI −0.80 to 1.13, *p* = 0.73). The results for neutralizing antibodies were very similar, with an increase in neutralizing capacity from the first to the second vaccination, a peak after the second vaccination and no major differences between the vaccinations with and without additional SARS-CoV-2 infection, as well as between vaccinated HCs and sAID patients (Figure 1B).

### 3.3. Relation between Anti-SARS-CoV-2 Antibody Levels and Neutralizing Capacity

We have also analyzed the association between anti-SARS-CoV-2 antibody levels and the neutralizing capacity. Figure 1C shows a descriptive plot showing the neutralizing antibody levels observed at each total antibody measurement. The regression lines fit separately for HCs and sAID patients, suggesting that at the lower range of antibody levels, HCs show higher levels of neutralizing capacity compared to sAID patients, and neutralizing capacity becomes similar with higher antibody levels. We have analyzed this relationship using a mixed-effects linear regression model with the logit-transformed neutralizing antibody levels as the dependent variable, log-transformed antibody levels and study groups as fixed effects and patient identifier and sample collection timepoint (i.e., vaccination or infection) as crossed random effects. By adding an interaction term between the study group and the log-transformed antibody levels, we tested the equality of the slopes between sAID and HC groups for the relationship between the antibody levels and neutralizing capacity. This interaction term was non-zero, indicating that the relationship between the total antibody levels and neutralizing antibody levels depended on the study group (*p* for interaction = 0.0051).

### 3.4. Association between Immunomodulatory Treatment and Vaccination Response

**Figure 2:**
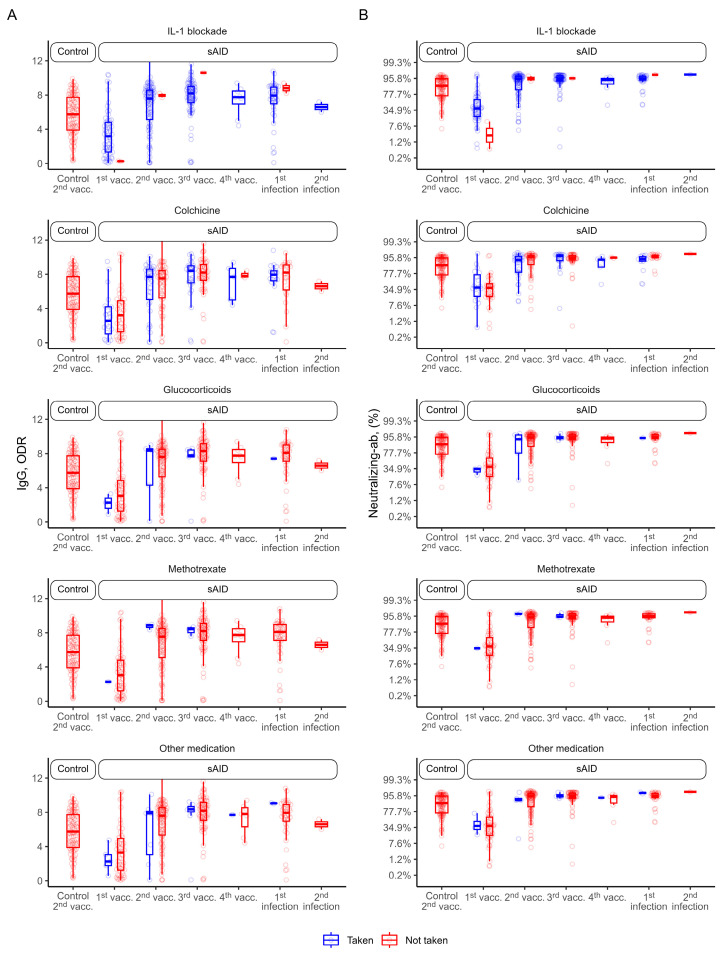
Distribution of antibody levels by type of treatment. “Not taken” here refers to the single drug considered. (**A**) Anti-SARS-CoV-2 IgG antibodies and (**B**) SARS-CoV-2 neutralizing antibodies for sAID patients and HCs after COVID-19 vaccination and disease. HC, healthy control; IgG (here), anti-SARS-CoV-2 IgG antibodies; Neutralizing-ab, neutralizing antibodies; ODR, optical density ratio; sAID, systemic autoinflammatory disease; vacc., vaccination.

We did not observe lower antibody levels in sAID patients compared to HCs, and since the vast majority of the sAID patients but none of the HCs received IL-1 inhibition, there seems to be no effect from IL-1 inhibitors on SARS-CoV-2 vaccination response. Accordingly, the few sAID patients who did not use IL-1 inhibitors did not show different vaccination responses compared to those using IL-1 inhibitors (Figure 2A,B). We next assessed whether additional therapies with colchicine, methotrexate or glucocorticoids affected vaccination responses in sAID patients but no major differences in anti-SARS-CoV-2 IgG antibodies or neutralizing antibodies were found. Only one patient receiving mycophenolate and tacrolimus treatment at different timepoints showed negative results in both ELISA tests at all time points.

## 4. Discussion

This study shows that SARS-CoV-2 vaccines trigger adequate humoral responses in sAID patients. Anti-SARS-CoV-2 IgG antibodies as well as neutralizing antibodies are already formed after primary vaccination and increase after the second vaccination to a level that is comparable to that of HCs. These results, acquired from a rather large sAID population treated with IL-1 inhibitors, confirm the initial data obtained in a smaller group of sAID patients [9].

One unexpected finding of this study was that antibody responses appeared to be even higher in sAID patients than in HCs. These differences, however, disappeared after adjusting for the time interval between vaccination and sample acquisition. On the other hand, we also found a slightly lower neutralizing antibody response in sAID patients for a given total antibody level at the lower range.

Whether vaccination protects sAID patients from severe COVID-19 is beyond the scope of our study. However, it was interesting to observe that a large proportion of sAID patients (79% of all infected persons) only contracted the virus during the “fifth Corona wave” (from calendar week 51/2021 on), when a majority of patients had already received triple vaccinations. The four unvaccinated patients only had low anti-SARS-CoV-2 IgG antibodies after infection. Whether a certain immunomodulatory treatment used for the treatment of sAIDs dampens vaccination responses is another important question: Our results suggest that therapy with an IL-1 inhibitor, which the vast majority of our patients received, did not have a negative impact on vaccination response. Also, further treatment with colchicine did not affect the vaccination response. Only one patient treated with a combination of mycophenolate showed low antibody titers across all measurement time points, which could be ascribed to the mycophenolate treatment as previously observed [10].

Our study has some limitations. Although the HC group had a similar age and sex distribution to the sAID group, we were only able to compare the sAIDs to HCs at different average time intervals after their second vaccination. We have addressed this issue with regression adjustments and observed that the apparently higher antibody levels in sAID patients were explained by this time difference in sample collection. Our findings on the non-uniform association between total and neutralizing antibody levels in controls vs. sAIDs could also have been affected for the same reason; therefore, this finding needs to be interpreted accordingly. In addition, we did not collect information on adverse events after vaccination as we focused on the analysis of antibody responses. However, a previous study on a large cohort of FMF patients presented reassuring results, as they did not report any relevant safety concern or an increasing number of flares after vaccination [11]. Finally, we did not have data on the persistence of the vaccination response in sAID patients.

## 5. Conclusions

Primary SARS-CoV-2 vaccination as well as booster vaccinations showed efficacy in sAID patients. In general, immunosuppressive therapy with IL-1 inhibitors could not be associated with a weakened immune response to the vaccination. One further research goal could be to investigate the long-term course of the antibody response. Hence, we do not see the necessity to control anti-SARS-CoV-2 antibody responses and neutralizing antibodies in sAID patients to ensure vaccination success.

The data provide the basis for recommendations and guidelines for the clinician in the consultation of sAID patients in vaccination planning.

## Figures and Tables

**Table 1:** Demographics and characteristics of patients and controls.

	sAID	HC
*n*	100	100
Demographic characteristics		
Age, years	42.7 ± 16.0	42.7 ± 15.8
Females, *n* (%)	58 (58.0)	57 (57.0)
Body weight	77.7 ± 21.5	-
Current smokers, *n* (%)	22 (22.0)	-
Comorbidities, *n* (%)		
Diabetes	10 (10.0)	-
Hypertension	23 (23.0)	-
History of CV event	8 (8.0)	-
History of thrombotic event	7 (7.0)	-
Type of sAID, *n* (%)		
FMF	37 (37.0)	0
AoSD	25 (25.0)	0
Gout	15 (15.0)	0
CAPS	7 (7.0)	0
sAID of unclear etiology	7 (7.0)	0
Behçet’s disease	4 (4.0)	0
TRAPS	4 (4.0)	0
Yao syndrome	1 (1.0)	0
COVID-19 vaccinations, *n* (%) *		
1st	96 (96.0)	100 (100.0)
2nd	95 (95.0)	100 (100.0)
3rd	82 (82.0)	-
4th	9 (9.0)	-
Immune-modulatory treatment, *n* (%)		
IL-1 inhibitors	98 (98.0)	0
Anakinra	48 (48.0)	0
Canakinumab	50 (50.0)	0
No IL-1 inhibitors	2 (2.0)	0
Colchicine	36 (36.0)	0
Glucocorticoids **	5 (5.0)	0
csDMARDs	4 (4.0)	0
MTX	3 (3.0)	0
Allopurinol	2 (2.0)	0
Febuxostat	2 (2.0)	0
COVID-19 infections, *n* (%)		
Total infected	43 (43.0)	-
Infected more than once	3 (3.0)	-

AoSD, Adult-onset Still’s disease; CAPS, cryopyrin-associated periodic syndrome; csDMARDs, conventional synthetic disease-modifying antirheumatic drugs; CV, cardiovascular; FMF, familial Mediterranean fever; HC, healthy control; IL, interleukin; MTX, methotrexate; sAID, systemic autoinflammatory disease; TRAPS, tumor necrosis receptor-associated periodic syndrome. * For sAID patients: The majority of patients had received a mRNA-based, not a vector-based vaccine––more specifically, 89% of all patients who received the 1st vaccination (85 patients with a mRNA-based vaccine and 11 with a vector-based vaccine), 96% (91 and 4) for the 2nd, 100% (82 and 0) for the 3rd and 100% (9 and 0) for the 4th vaccination. ** Overall, five patients used glucocorticoids, for only one of whom the dose was >10 mg.

**Table 2:** Mean anti-SARS-CoV-2 IgG and neutralizing antibodies.

		Anti-SARS-CoV-2 IgG		Neutralizing Antibodies in %	
Event	Sex	sAID	HC	sAID	HC
1st vacc.	F	3.3 ± 2.9 (*n* = 37) *	-	42.5 ± 30.2 (*n* = 32)	-
	M	3.4 ± 2.3 (*n* = 23)	-	40.2 ± 25.5 (*n* = 20)	-
	All	3.4 ± 2.7 (*n* = 60)	-	41.6 ± 28.2 (*n* = 52)	-
2nd vacc.	F	7.2 ± 2.2 (*n* = 56)	6.2 ± 2.2 (*n* = 57)	89.9 ± 15.5 (*n* = 54)	86.5 ± 17.6 (*n* = 57)
	M	6.0 ± 3.2 (*n* = 38)	5.1 ± 2.5 (*n* = 43)	77.2 ± 30.1 (*n* = 33)	77.3 ± 21.3 (*n* = 43)
	All	6.7 ± 2.7 (*n* = 94)	5.7 ± 2.4 (*n* = 100)	85.1 ± 22.9 (*n* = 87)	82.5 ± 19.7 (*n* = 100)
3rd vacc.	F	8.1 ± 1.7 (*n* = 45)	-	94.6 ± 5.3 (*n* = 43)	-
	M	7.2 ± 2.9 (*n* = 35)	-	85.3 ± 27.5 (*n* = 33)	-
	All	7.7 ± 2.3 (*n* = 80)	-	90.5 ± 19.1 (*n* = 76)	-
4th vacc.	F	7.3 ± 2.2 (*n* = 3)	-	92.4 ± 5.7 (*n* = 2)	-
	M	7.4 ± 1.7 (*n* = 5)	-	86.1 ± 20.6 (*n* = 5)	-
	All	7.4 ± 1.8 (*n* = 8)	-	87.9 ± 17.2 (*n* = 7)	-
1st infection	F	7.4 ± 2.4 (*n* = 29)	-	92.9 ± 9.8 (*n* = 23)	-
	M	7.3 ± 2.8 (*n* = 13)	-	90.7 ± 14.1 (*n* = 12)	-
	All	7.3 ± 2.5 (*n* = 42)	-	92.1 ± 11.3 (*n* = 35)	-
2nd infection	F	6.6 ± 0.8 (*n* = 2)	-	97.3 ± NA (*n* = 1)	-
	M	-	-	-	-
	All	6.6 ± 0.8 (*n* = 2)	-	97.3 ± NA ** (*n* = 1)	-

F, female; HC, healthy control; M, male; *n*, number; NA, not available; sAID, systemic autoinflammatory disease; vacc., vaccination. * The numbers (*n*) represent the number of results we have measured for each category, not necessarily the total number of individuals with 1st/2nd/3rd/4th vaccination or 1st/2nd infection in our study. ** We only had a serum from one patient to measure the neutralizing antibodies after 2nd infection, so there is no standard deviation (SD) here.

## Data Availability

All relevant data can be found in the manuscript.
